# Enhancing the Unicompartmental Knee Arthroplasty Safety via Finite Element Analysis of Coronary Plane Alignment: A Case Report

**DOI:** 10.7759/cureus.61765

**Published:** 2024-06-05

**Authors:** Takaaki Imada, Mitsuru Hanada, Kohei Murase, Yukihiro Matsuyama

**Affiliations:** 1 Department of Orthopaedic Surgery, Hamamatsu University School of Medicine, Hamamatsu, JPN; 2 Center for Industry-University Collaboration, Graduate School of Engineering Science, Osaka University, Osaka, JPN

**Keywords:** joint line, load position, finite element analysis, unicompartmental knee arthroplasty, knee osteoarthritis

## Abstract

Although Oxford unicompartmental knee arthroplasty is often used to successfully treat patients with knee osteoarthritis isolated at the medial compartment, we present a case of fracture just below the tibial keel caused by either a shift in medial loading position or an increased amount of tibial osteotomy. Finite element analysis was used to determine which factor was more important. First, a 3D-surface model of the patient’s tibia and the implant shape were created using computed tomography-Digital Imaging and Communications in Medicine (CT-DICOM) data taken preoperatively. The finite element analysis found that following unicompartmental knee arthroplasty, the cortical stress (normal, 5.8 MPa) on the medial tibial metaphyseal cortex increased as the load point moved medially (3 and 12 mm medially: 7.0 and 10.7 MPa, respectively) but was mild with increased tibial bone resection (2 and 6 mm lower: 6.1 and 6.5 MPa, respectively). Implanting the femoral component more medially than the preoperative plan increases stresses in the medial cortex of the tibia and may cause fractures.

## Introduction

Knee osteoarthritis (KOA) adversely affects the daily living of older people. The lifetime risk of KOA occurrence is reported to be 45% worldwide [1]. Unicompartmental knee arthroplasty (UKA) is the preferred treatment of KOA isolated to either the medial or lateral compartment, as it is less invasive than total knee arthroplasty (TKA) [2,3]. UKA is considered to cause a quick recovery in older patients and a high success rate [4-7]. Many previous reports of tibial medial condyle fracture intraoperatively or after UKA exist [8,9]. However, only a few have specifically quantified the risk of fracture.

Here, we report a case in which the primary reason for tibial medial condyle fracture after UKA was investigated using finite element analysis.

## Case presentation

**Figure 1:**
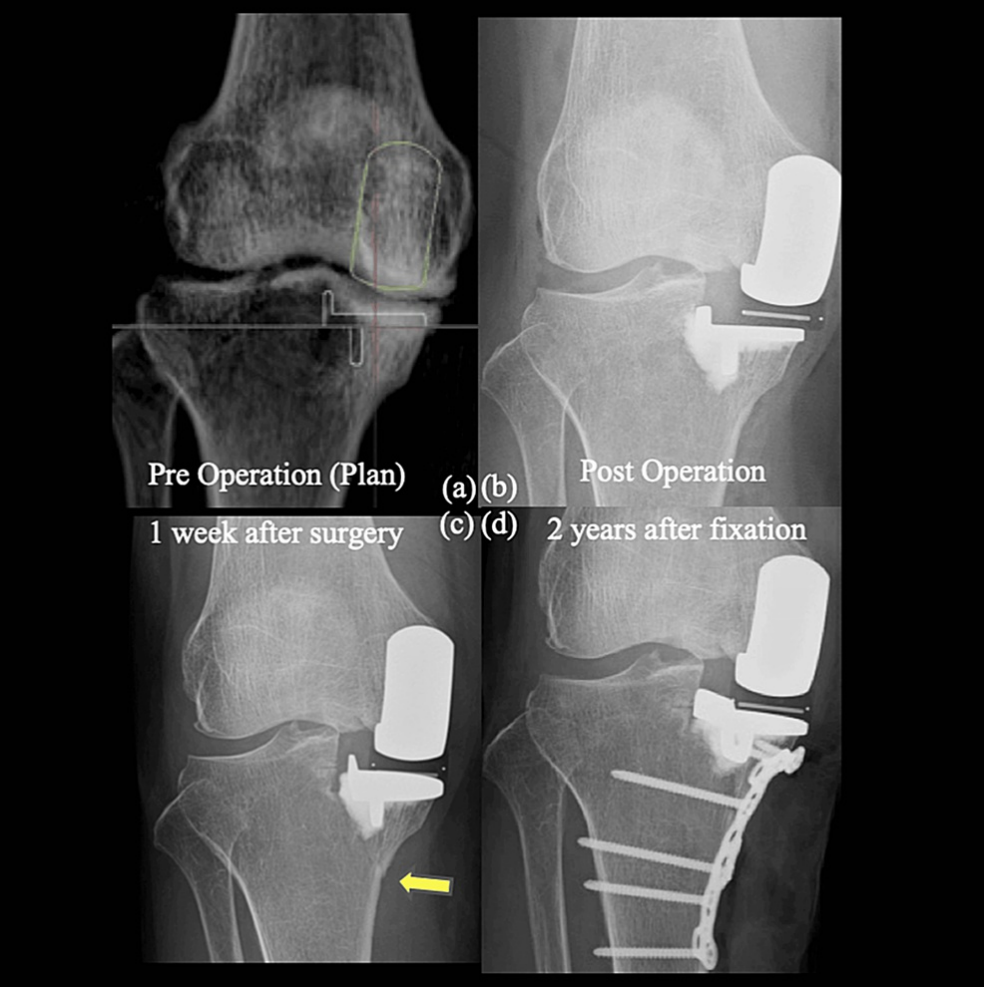
Preoperative implant placement plan and postoperative X-rays (a) Preoperatively simulated the implant placement position. (b) Postoperative implant placement position. (c) The postoperative one-week radiograph. (d) Radiograph two years after fixation surgery. Postoperatively, the tibial implant was 4 mm lower in the joint cut line than planned, and the femoral implant was placed 6 mm medial to the tibia. The postoperative one-week radiograph showed a medial tibia plateau fracture just below the keel. The two-year postoperative radiograph showed no subsidence of the tibial implant due to the proximal tibial plate.

An 83-year-old female underwent UKA to treat her right medial compartment KOA. Using preoperative computed tomography (CT) data and a three-dimensional (3D) template system for knee replacement surgery (ZedKnee: Lexi, Tokyo, Japan), we preoperatively simulated the implant placement position, size, and osteotomy amount. We determined the tibial implant was size A, with a 0° varus in the coronal plane and a 5° posterior tilt in the sagittal plane based on the tibial mechanical axis. Operatively, the tibial implant was the same size and was implanted in sagittal alignment according to the preoperative plan. However, the implant placement was malaligned in the coronal plane. The tibial medial condyle was resected 4 mm lower than that of the preoperative plan, and the femoral component was implanted at a site 6 mm medial to the standard. On postoperative day 1, the patient had permission to begin range of motion rehabilitation and ambulation. However, she experienced knee pain during rehabilitation one week postoperatively. Knee radiographs on day 7 revealed a medial tibia plateau fracture below the keel of the tibial component. Internal fixation with a proximal tibial plate was performed on day 10. The patient received teriparatide therapy and was instructed to avoid weight-bearing for four weeks following the additional surgery. Two years have passed since the surgery, and the patient is able to walk without knee pain (Figure 1).

The low joint cut line of the tibial medial condyle and the medial position of the femoral component may have affected the tibial plateau fracture. We used finite element analysis to determine whether the medial position of the femoral component or the amount of tibial medial condyle osteotomy significantly impacts tibial plateau fracture after UKA.

**Figure 2:**
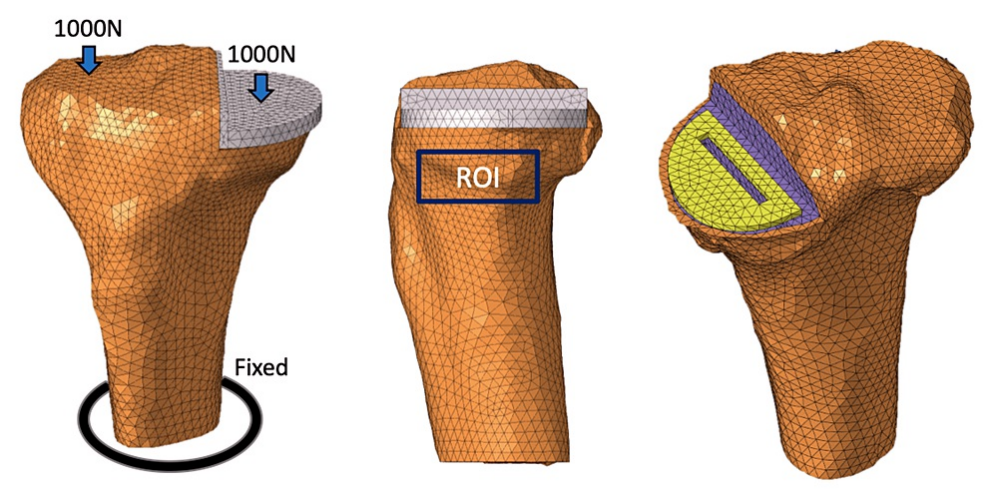
Unicompartmental knee arthroplasty (UKA) load conditions A total of 1000 N was added to the medial and lateral tibial articular surfaces parallel to the tibial axis. The tibial diaphysis was fixed. Equivalent stresses were quantitatively measured in the region of interest (ROI) of the proximal tibia.

**Figure 3:**
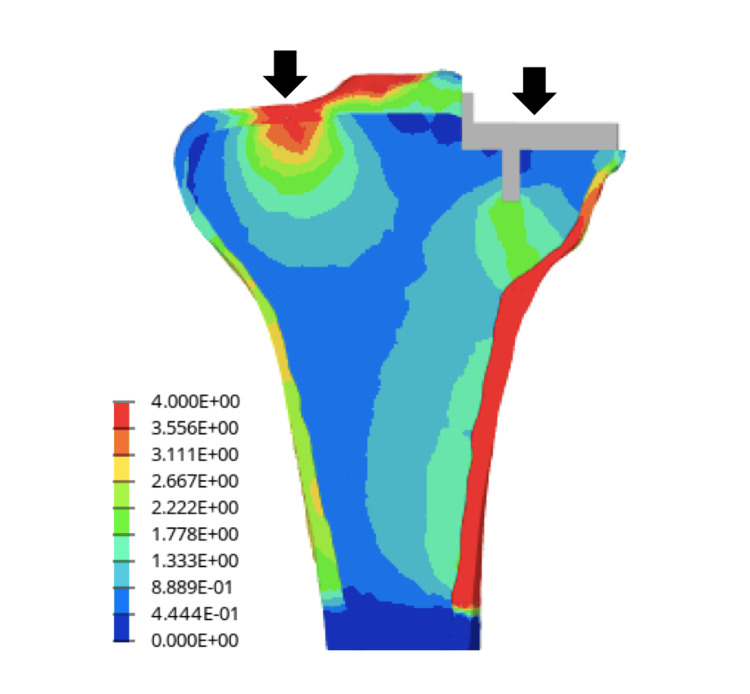
von Mises stresses in the cancellous bone in the coronal section The von Mises stress values were concentrated in the medial metaphyseal cortex of the tibia from below the keel. It was similar to the fracture line.

**Figure 4:**
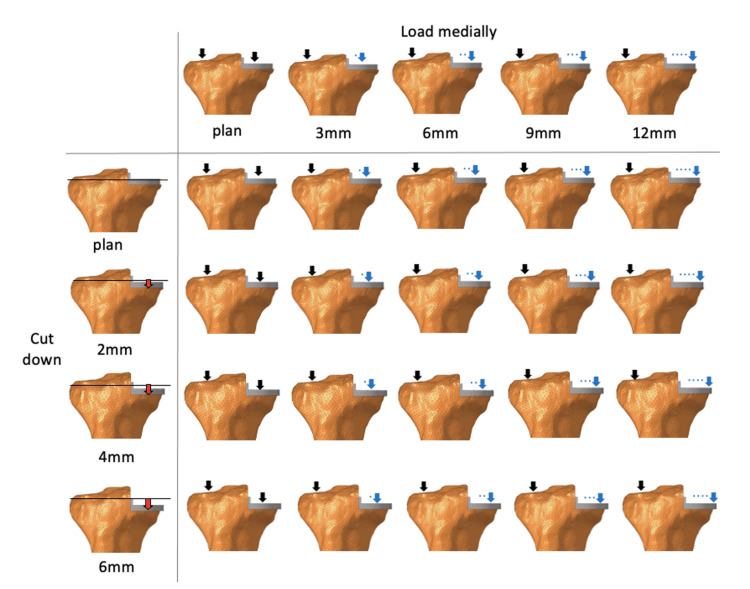
Increased osteotomy and changes in load position The figure simulates where the load position was moved medially and tibial osteotomy was increased.

**Figure 5:**
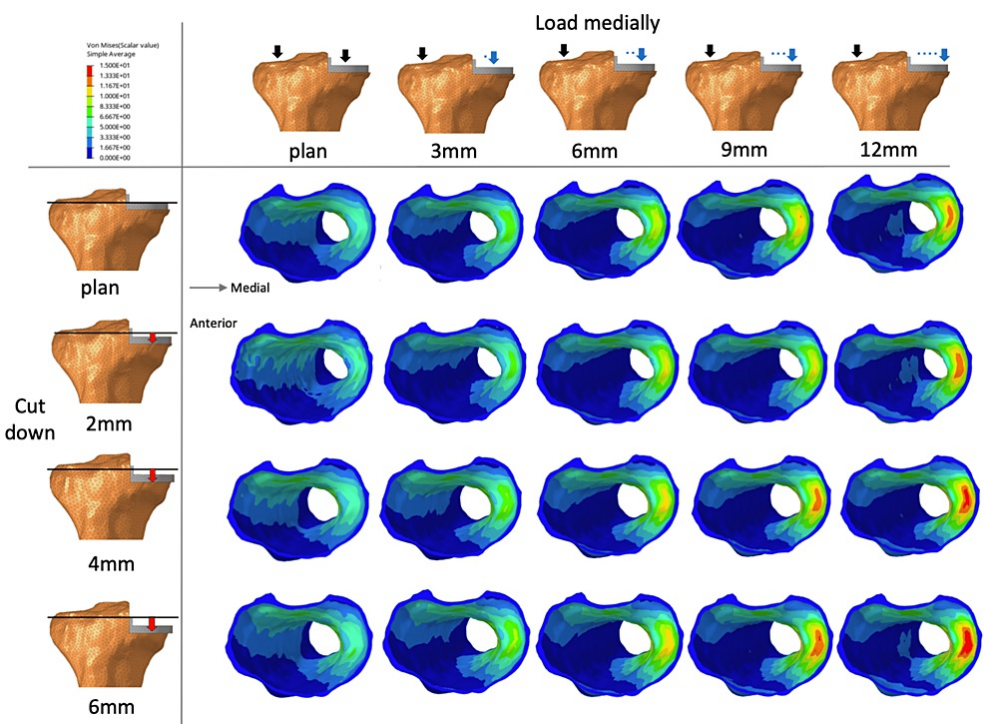
von Mises stresses in the medial cortical bone von Mises stresses in the medial cortical bone of the tibia in the axial section when the load position moves medially and the articular surface is lowered.

**Figure 6:**
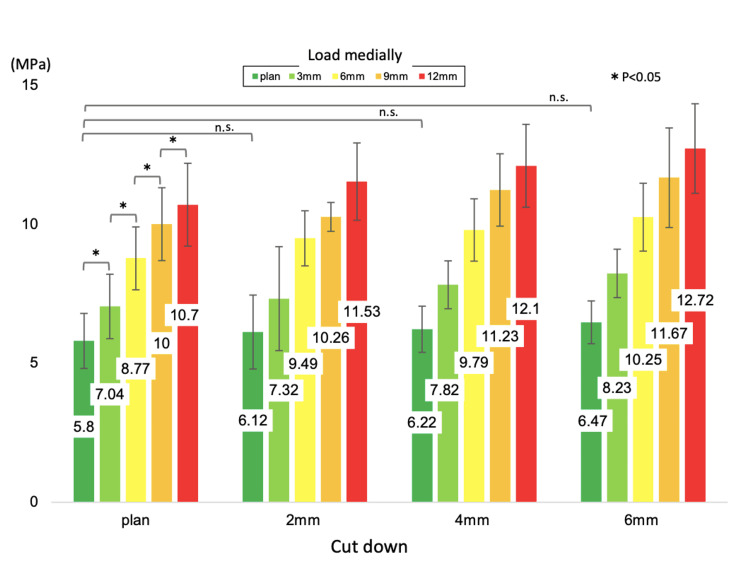
The mean value of von Mises stresses in the region of interest (ROI) The mean value of von Mises stresses in the ROI when the load position moves medially and the articular surface is lowered. As the loading point is moved medially by 3 mm, the von Mises stress values increase, with significant differences at each point.

A 3D surface model including the cancellous and cortical bones of the tibia was constructed from preoperative CT data (per 1 mm slice) of patients with anteromedial KOA using 3D model software (Mimics Ver.14. Materialise, Yokohama, Japan) [10-12]. Subsequently, the model was divided into multi-elements using finite element modeling software (Computer Aided Design (CAD): HyperWorks, Michigan, USA). Finally, a finite element model consisting of 171,637 secondary elements of the tetrahedron was constructed. The tibial component was also positioned from preoperative CT data using ZedKnee (Lexi, Tokyo, Japan). A CAD was used to model the same size as the positioned Oxford UKA size A (anterior-posterior diameter of 45.2 mm, mediolateral width of 26.0 mm). The tibial component position was defined as 0° varus in the coronal plane, 5° posterior tilt in the sagittal plane, and neutral rotation according to Akagi's line. Cement thickness was set at 1 mm. The femoral and tibial components were fully bonded to the femur and tibia, respectively, simulating the use of cement. The medial intercondylar spine acted as an anatomical landmark for the vertical cut. The tibial diaphysis was fixed 90 mm distal to the medial intercondylar spine [13]. Assuming the weight of the patient (approximately 40 kg) and the load on the knee when standing on one leg (approximately five times the body weight), 1000 N was added to each of the medial and lateral tibial articular surfaces parallel to the tibial axis [14]. The lateral load point was defined as the middle point of the lateral femoral condyle, and the medial loading point was defined as the tibial component surface relative to the center of the femoral component [15]. Each material is assumed to be a linear elastic body. Table 1 shows each material constant (Young's modulus and Poisson's ratio) [13,16]. We defined the region of interest (ROI) on the proximal tibia for the quantitative equivalent stress as 15 mm below the tibial medial condylar articular surface of the intact knee [14] (Figure 2). The nonlinear structural finite element analysis was performed using LS-DYNA (ANSYS Corporation, Pennsylvania, USA) to evaluate tibial deformation due to shear forces using von Mises stress values. Finite element analysis revealed that the distribution of equivalent stress (von Mises) values in the tibia occurred in the central coronal plane of the keel. The von Mises stress values were concentrated below the keel to the medial metaphyseal cortex of the tibia and appeared to be the fracture line (Figure 3). Additionally, the von Mises stress values occurring in the medial tibial cortex were measured when the loading position was moved medially by 3 mm and when the amount of tibial osteotomy was increased by 2 mm (Figure 4). As the load point moved medially, the von Mises stress values generated in the cortex just below the implant increased. In contrast, increasing the amount of tibial osteotomy did not result in a sudden increase in stress in the tibial metaphyseal cortex (Figure 5). As the load point moved by 3 mm medially from the reference point, the von Mises stress values increased by 21-84% from the reference point (normal: 5.8, 3 mm medially: 7.0, 6 mm medially: 8.8, 9 mm medially: 10.0, 12 mm medially: 10.7 (MPa)). In contrast, as the tibial osteotomy was increased by 2 mm from the reference position, the von Mises stress values at the fracture site increased only by 7-12% (normal: 5.8, 2 mm lower: 6.1, 4 mm lower: 6.2, 6 mm lower: 6.5 (MPa)) (Figure 6).

**Table 1:** Material property CoCrMo ally: cobalt-chromium-molybdenum ally

Material	Young’s modulus (MPa)	Poisson’s ratio
Cortical bone	5000	0.3
Cancellous bone	1000	0.25
Cement	500	0.3
CoCrMo ally	200000	0.3

## Discussion

The global number of cases treated with UKA has increased in recent years, given that the Oxford UKA procedure is less surgically invasive, preserves ligaments to maintain physiological rotation, and promotes earlier rehabilitation than TKA [17]. However, reports of tibial medial condyle fractures have also increased [8], most occurring during the perioperative period and related to procedural errors during surgery [9].

The loading position in the present case was shifted 6 mm medial and 4 mm inferior to the preoperative plan. Based on the finite element analysis, the stress on the medial cortex of the tibia increased by 51% due to the 6 mm medial shift of the loading position and by 7% due to the 4 mm downward shift of the loading position. In other words, the increased stress in the medial tibial cortex was caused more by the medial shift than the downward shift of the load position.

A previous study using finite element analysis reported that the stress applied to the medial tibial cortex following UKA increased by 24.6% compared to that before surgery and that the stress increased rapidly when the loading position on the tibia shifted medially by more than 3 mm [16]. Zhu et al. also reported increased stress in the medial tibial cortex when the bearing is placed away from the tibial tray wall by a femoral implant [14]. Our analysis revealed similar results. When the load point shifted medially, the distance between the vertical osteotomy line and the load point increased, leading to an expected increase in the shearing force from the keel to the medial metaphyseal cortex of the tibia.

As reported previously, the causes of medial tibial condyle fracture include tibial implant valgus placement, size mismatch, medial condyle injury due to osteotomy guide fixation pin, posterior tibial cortical injury during vertical tibial osteotomy, and tibial implant pressing [9]. However, no case report has reported that the position of femoral implant placement influences fracture, and the causal relationship with clinical results is poorly understood [18]. The present study suggests that implanting the femoral component more medially than in the preoperative plan increases stresses in the medial cortex of the tibia and may cause fractures.

This study had a few limitations. First, the data is from a single patient, and the figures derived may vary depending on the patient’s sex, height, weight, and tibial bone morphology [19]. Second, this study excludes the effects of the soft tissues around the knee (ligament, meniscus, muscle). For the hip joint, muscle strength plays an important role in the measured peri-hip strain [20]. However, a UKA periprosthetic-only study reported that omitting the muscle forces acting at the tibial attachment site did not significantly affect the results [16]. Third, the fracture also could have been caused by the impaction process of implanting the cemented tibial baseplate. The study only assessed the malalignment of the implants and did not examine the possibility of intraoperative fractures occurring.

## Conclusions

A finite element analysis of the medial tibial condyle of Oxford UKA was performed. von Mises stress was concentrated from just below the keel to the medial metaphyseal cortex of the tibia, and it increased as the load point moved medially. These findings provide valuable insight to surgeons regarding the prevention of future fractures in such patients.
